# *Hoxa5*: A Key Player in Development and Disease

**DOI:** 10.3390/jdb4020013

**Published:** 2016-03-25

**Authors:** Lucie Jeannotte, Florian Gotti, Kim Landry-Truchon

**Affiliations:** 1 Centre de recherche sur le cancer de l′Université Laval; CRCHU de Québec, L′Hôtel-Dieu de Québec, QC G1R 3S3, Canada; florian.gotti.1@ulaval.ca (F.G.); kim.landry-truchon.1@ulaval.ca (K.L.-T.); 2 Department of Molecular Biology, Medical Biochemistry and Pathology, Université Laval, QC G1V 0A6, Canada

**Keywords:** *Hox* genes, organogenesis, tumorigenesis, gene regulation, long non-coding RNA

## Abstract

A critical position in the developmental hierarchy is occupied by the *Hox* genes, which encode transcription factors. *Hox* genes are crucial in specifying regional identity along the embryonic axes and in regulating morphogenesis. In mouse, targeted mutations of *Hox* genes cause skeletal transformations and organ defects that can impair viability. Here, we present the current knowledge about the *Hoxa5* gene, a paradigm for the function and the regulation of *Hox* genes. The phenotypic survey of *Hoxa5^−/−^* mice has unveiled its critical role in the regional specification of the skeleton and in organogenesis. Most *Hoxa5^−/−^* mice die at birth from respiratory distress due to tracheal and lung dysmorphogenesis and impaired diaphragm innervation. The severity of the phenotype establishes that *Hoxa5* plays a predominant role in lung organogenesis *versus* other *Hox* genes. *Hoxa5* also governs digestive tract morphogenesis, thyroid and mammary glands development, and ovary homeostasis. Deregulated *Hoxa5* expression is reported in cancers, indicating *Hoxa5* involvement in tumor predisposition and progression. The dynamic *Hoxa5* expression profile is under the transcriptional control of multiple *cis*-acting sequences and *trans*-acting regulators. It is also modulated by epigenetic mechanisms, implicating chromatin modifications and microRNAs. Finally, lncRNAs originating from alternative splicing and distal promoters encompass the *Hoxa5* locus.

## 1. Introduction

Embryonic development can be viewed as a hierarchy of molecular events controlled by the concerted action of regulatory networks. *Hox* genes occupy a central position in the control of the formation of body segment-specific structures by regulating the transcription of downstream effectors that, in turn, direct the morphogenetic events leading to complex body forms [1]. Consequently, mutations in *Hox* genes alter segmental identity and cause morphological defects [2]. In human and mouse, 39 *Hox* genes are distributed over four complexes located on different chromosomes. They are expressed sequentially in time and space according to their position in the complexes. Accordingly, the 3′ most genes are expressed earlier and in more anterior domains along the embryonic axes than the 5′ located ones [3]. This clustered organization is fundamental for the precise spatio-temporal regulation and the function of each gene and hence for the correct patterning of the embryo [4,5]. Different *Hox* genes are expressed in discrete but overlapping domains along the developing anterior-posterior axis. A specific combination of HOX proteins at a particular anterior-posterior level thus provides a unique genetic address that determines the structures at this level. Analyses of *Hox* mutant mice phenotypes endorse the collinear relationship existing between the position of individual genes within the *Hox* clusters and the structural defects observed along the anterior-posterior axis.

Based on sequence homology and their relative position within the cluster, *Hox* genes are subdivided into 13 paralog groups. The similarities in protein structure and expression pattern among *Hox* paralogs indicate that they can perform overlapping functions. Indeed, compound mutant mice for *Hox* paralogs exhibit new or more severe phenotypes than single *Hox* mutant mice [6]. Furthermore, knock-in substitutions of *Hox* genes by their paralogs confirm that they can fulfill similar roles [7,8]. 

*Hox* genes encode transcription factors that bind via their homeodomain DNA motifs in HOX-responsive elements. Despite similar DNA-binding specificity *in vitro*, individual HOX proteins confer different regulatory actions *in vivo*. This differential selectivity is largely achieved by their interaction with cofactors, mainly members of the PBC and MEIS homeodomain protein families [9,10]. While data on the developmental role of *Hox* genes are rapidly expanding, understanding how *Hox* genes regulate regional identity and organogenesis remains an issue and still awaits the identification of target genes. So far, HOX proteins have been shown to control the expression of other "high-level executive" genes encoding transcription factors, including *Hox* genes, morphogen signals, as well as effectors mediating cell behavior [11]. Not surprisingly, mutations or inappropriate expression of *Hox* genes can disrupt normal programs of growth, leading to various genetic disorders and diseases, including cancer [12,13].

The *Hoxa5* (initially named *Hox1.3*) gene belongs to this large gene family. It is positioned near the middle of the *HOXA* cluster located on mouse chromosome 6 and human chromosome 7. *Hoxa5* encodes a 270 amino acid ANTP-class homeodomain protein [14].

## 2. *Hoxa5*: An Imperative in Morphogenesis

Analysis of *Hox* mutant mice has revealed a plethora of phenotypes including skeletal homeotic transformations, organ defects, and postnatal phenotypes that are indicative of the broad range of actions of *Hox* genes throughout life. The same situation prevails for the *Hoxa5* gene as revealed by the phenotypic survey of *Hoxa5* mutant mice, which unveiled the importance of *Hoxa5* in the development of several tissues and organs (Table 1). Moreover, the loss of *Hoxa5* function is among the few single *Hox* gene mutations that cause death at birth.

### 2.1. Axial Skeleton

As most *Hox* mutants, *Hoxa5* null mutants present homeotic transformations of the axial skeleton. They are essentially localized in the cervical and upper thoracic regions, between the third cervical (C3) and second thoracic (T2) vertebrae [15,16]. The anterior transformation of C6 into the likeness of C5 with the loss of the tuberculum anterior and the posterior transformation of C7, which adopts the identity of a thoracic vertebra with the presence of ectopic ribs, are the most penetrant skeletal anomalies observed. They are present in all *Hoxa5^−^*^/*−*^ mutants from the different genetic backgrounds tested. Other skeletal malformations are also detected at a lesser prevalence. They include the presence of an ectopic dorsal process on T1, which then resembles T2, and the loss or the reduction of the dorsal process normally found on T2 [16]. *Hoxa4* and *Hoxa6* single mutants also present homeotic transformations in the cervicothoracic transition region but to a smaller extent [17,18]. However, these shared phenotypes appear to be due to the loss of *Hoxa5* expression in the C3–T2 region of the prevertebral column in *Hoxa4* and *Hoxa6* mutant embryos resulting from deleterious long-range *cis* effects of the *neo* cassettes inserted into the *Hoxa4* and *Hoxa6* alleles that hinder the transcription from the *Hoxa5* promoter [16,19]. Taken together, these data establish the importance of *Hoxa5* in the patterning of the skeletal cervicothoracic region.

### 2.2. Appendicular Skeleton

Besides transformations of the axial skeleton, the *Hoxa5* mutation affects the appendicular skeleton, more specifically the acromion, a digit-like projection emerging from the spina scapula of the pectoral girdle to articulate with the clavicula [16]. Depending on the genetic environment, *Hoxa5* mutants present a reduced, interrupted or missing acromion. This defect is also seen in *undulated* (*un*) mice, which carry a point mutation in the *Pax1* gene [32,33,34]. *Hoxa5* and *Pax1* genes are coexpressed over a large domain along the developing vertebral column but they show a genetic cooperation at the cervicothoracic transition level where the pectoral girdle is aligned. The expressivity and the penetrance of the skeletal anomalies affecting C6, T1 and T2 are augmented when one mutant allele is introduced into the other mutant background [20]. In acromion formation, *Hoxa5* and *Pax1* act in a complementary way: *Hoxa5* provides regional cues for the correct onset of *Pax1* expression in the developing pectoral girdle, while *Pax1* promotes the recruitment of acromion precursor cells. Whether HOXA5 acts directly on *Pax1* expression remains to be elucidated. *Hoxa5* is also involved in the control of chondrogenesis by negatively regulating the expression of *Sox9*, a master regulator of cartilage development [35]. The negative action of *Hoxa5* on *Sox9* expression was reported in chick somites suggesting that it may constitute an evolutionary-conserved pathway used by *Hoxa5* to regulate cartilage development and morphology [36]. 

Mutant mice for the *Hoxb5* paralog gene also present anomalies in the axial and appendicular skeletons, distinct from those observed in *Hoxa5* mutants but covering a similar territory [37]. These observations add the concept of regional functional complementarity of *Hox* genes to the frequently evoked notion of functional redundancy [38].

### 2.3. Respiratory System

The *Hoxa5* null mutation distinguishes itself by a high mortality rate at birth of the *Hoxa5* homozygous mutant pups (*Hoxa5^−/−^)*, whereas heterozygous mutants (*Hoxa5^+/−^*) survive with a normal lifespan and no obvious phenotype [15]. *Hoxa5^−/−^* newborns die from respiratory distress due to severe alterations of the respiratory tract, a phenotype that is coherent with the strong *Hoxa5* mesenchymal expression along the entire respiratory system [21,22]. 

Laryngotracheal malformations are fully penetrant in *Hoxa5^−^*^/*−*^ mice. The phenotype includes an important reduction of the luminal surface of the trachea, a profound disorganization of the epithelium and a thickening of the lamina propria with the formation of polyp-like structures. In *Hoxa5^−^*^/*−*^ mice, the C-shaped cartilage rings, which normally encircle the ventrolateral surfaces of the trachea and primary bronchi to prevent collapse during respiration, present ventral gaps along the trachea, a reduced number of rings and an abnormal banding pattern. Moreover, the cartilage does not extend as dorsally as in controls. In the worst cases, a near-complete tracheal occlusion occurs contributing to the death of *Hoxa5^−^*^/*−*^ pups at birth. This phenotype is reminiscent of human tracheal stenosis [21,22,39]. The tracheal malformations are similar to those observed in mutant mice for the *Fstl1* gene, which encodes a bone morphogenetic protein (BMP) antagonist [40]. *Fstl1* expression is reduced in the upper airways of *Hoxa5* mutants and ChIP experiments pinpointed *Fstl1* as a HOXA5 transcriptional target [22].

Profound anomalies affect the developing lungs of *Hoxa5* mutant embryos. They include the disorganization of the lung mesenchyme during early lung development and reduced branching morphogenesis that affects the subsequent formation of the saccula and results in an abnormal compact lung appearance with narrower airspaces and thicker mesenchyme prior to birth [21]. Decreased lung branching together with reduced cell proliferation also contribute to lung hypoplasia in *Hoxa5* mutant embryos, a phenotype that is rescued in surviving mutants by the time they are weaned [22,23]. The expression of the *Nkx2-1* and *Foxa2* genes is diminished in the lung epithelium of *Hoxa5*^−/−^ embryos [21]. The NKX2-1 and FOXA2 transcriptional factors are known to govern lung epithelial cell differentiation and to regulate the expression of surfactant proteins [41,42]. Accordingly, *Hoxa5* mutant collapsed lungs present decreased surfactant protein expression. As well, lung epithelial cell fate is perturbed in *Hoxa5* mutants. A reduced number of secretory club (Clara) cells is displayed in upper airways and less alveolar type I pneumocytes, which are normally in close association with vascular endothelial cells for gas exchange, are detected in the distal lung epithelium [22,24]. As *Hoxa5* expression is restricted to the lung mesenchyme, the epithelial alterations seen in *Hoxa5^−^*^/*−*^ lungs imply that HOXA5 acts indirectly on the respiratory epithelium. 

*Hoxa5* has also cell-autonomous functions. HOXA5 protein expression is detected in alveolar myofibroblast progenitors as shown by its colocalization with GFP from the *GFP* knock-in allele at the *Pdgfr* locus [23]. Alveolar myofibroblasts are interstitial contractile cells responsible for elastin deposition and alveolar formation, two processes affected in *Hoxa5^−^*^/*−*^ mutants. Indeed, the motility of alveolar myofibroblast precursors is impaired in *Hoxa5* mutants. This leads to the mispositioning of alveolar fibroblasts that become confined to the alveoli parenchyma, which causes elastic fiber disorganization and altered alveogenesis [23,43]. Moreover, *Hoxa5* mutants show an undeveloped lung microvasculature characterized by fewer endothelial cells trapped within the dense mesenchyme, a phenotype that is consistent with *Hoxa5* expression in the lung endothelium [22]. Thus, *Hoxa5* can act directly on alveolar myofibroblast progenitors and endothelial cells, but necessitates mesenchymal-epithelial communication to control lung epithelial processes like branching and cell fate specification.

A small proportion of *Hoxa5^−^*^/*−*^ mice survives and reaches adulthood. Surviving *Hoxa5* mutants develop an emphysema-like phenotype characterized by lung airspace enlargement, as mentioned above. The decreased lung surface area available for gas exchange and the increased upper airway resistance due to tracheal obstruction perturb the respiration of *Hoxa5* mutants. To compensate for these morphological defects, *Hoxa5^−^*^/*−*^ mice present a higher breathing frequency and overall minute ventilation in resting conditions. When facing hypoxia, *Hoxa5^−^*^/*−*^ mice adapt their tidal volume and breathing frequency to maintain a higher minute ventilation [26]. Thus, *Hoxa5* mutants develop breathing strategies to counteract their deficit in gas exchange capacity.

*Hoxa5^−^*^/*−*^ mice also present mucus hypersecretion caused by goblet cell metaplasia [23]. The latter results from the transdifferentiation of club cells into goblet cells, an event that initiates prior to birth [24]. Goblet cell metaplasia in *Hoxa5* mutants is accompanied by increased expression of the SPDEF and FOXA3 transcription factors that govern goblet cell specification [44,45]. Goblet cell metaplasia is often associated with the loss of FOXA2 expression in airway epithelium [46,47]. However in *Hoxa5* mutants, the process is FOXA2-independent, a result analogous to that observed when Notch signaling is activated in the lung epithelium [48]. Similarly, an increased Notch signaling activity occurs in the lung airway epithelium from *Hoxa5^−^*^/*−*^ mice and in areas of goblet cell metaplasia in patients suffering from chronic obstructive pulmonary disease (COPD) [24]. Treatment of *Hoxa5^−^*^/*−*^ mice with a γ-secretase inhibitor blocking Notch signaling attenuates the goblet cell metaplasia phenotype, pinpointing a potential therapy to inhibit mucus overproduction in human airway diseases. Thus, the loss of *Hoxa5* function in the lung mesenchyme impacts on epithelial cell fate by modulating Notch signaling in the lung epithelium. Studies of a mouse line carrying mutations in all three *Hox5* paralog genes show that the Wnt canonical signaling pathway also mediates *Hox5* mesenchyme-to-epithelium action in the lung [49]. The strong *Hoxa5* expression in the developing lung and the severity of the *Hoxa5* lung phenotype *versus* other single *Hox* mutant mice suggest that *Hoxa5* plays a predominant role in the combinatorial control of signaling pathways during lung formation [22,50].

*Hoxa5* is expressed in the phrenic motor column, a group of motor neurons that provides the exclusive source of diaphragm innervation [25]. In agreement, *Hoxa5^−^*^/*−*^ mutants present innervation defects of the diaphragm, which likely participate to the lung hypoplasia phenotype as lung morphogenesis is influenced by physical forces such as fetal breathing movements [22,51,52].

In summary, *Hoxa5* contributes extensively to lung development, maturation and function as revealed by the severe anomalies that affect the respiratory system of *Hoxa5^−^*^/*−*^ mice. These phenotypes are in agreement with the strong expression of the *Hoxa5* gene along the entire respiratory track mesenchyme and in the phrenic motor neurons responsible for diaphragm innervation. Interestingly, several *Hoxa5* phenotypes share characteristics with human pathologies. As well, changes in *HOXA5* expression are associated with pulmonary diseases. For instance, *HOXA5* expression is altered in patients suffering from developmental diseases such as bronchopulmonary dysplasia, and in adult diseases like lung cancer, primary lung hypertension and COPD, one of the leading mortality causes worldwide [53,54,55]. Accumulated evidences on the mechanisms contributing to adult lung pathogenesis support the notion that genetic alterations affecting lung developmental processes in early life may later function as a trigger for an apparently adult onset of lung diseases [56]. The results accumulated so far indicate that deciphering the developmental role of the *Hoxa5* gene may contribute resolving the molecular mechanisms underlying lung pathologies.

### 2.4. Digestive System

The loss of *Hoxa5* function also affects gut morphogenesis. In mice, the gut derives from two endodermal folds, the anterior and posterior intestinal portals that fuse ventrally and join at the yolk stalk level. The oesophagus and the stomach both originate from the foregut, while the midgut develops into the gastrointestinal tract and the hindgut forms the colon [57,58]. *Hoxa5* is expressed in a dynamic spatio-temporal fashion during gut formation [15,27,28,59,60]. *Hoxa5* transcripts are first detected at embryonic (E) day 9 in the primitive gut mesenchyme. In the prospective stomach, *Hoxa5* expression presents a rostro-caudal gradient from E12.5 to E17.5. Then, *Hoxa5* transcripts become mainly confined to the submucosal and muscular layers of the hindstomach. No *Hoxa5* expression is detected in the stomach after weaning age. In the midgut, *Hoxa5* expression becomes restricted to the myenteric plexus of the enteric nervous system around E14.5, a pattern maintained up to adulthood. A similar situation prevails in the hindgut. Coherent with the *Hoxa5* expression along the gut, the loss of *Hoxa5* function affects the entire gastrointestinal tract. In absence of *Hoxa5* function, goblet cells are abnormally distributed along the colon [27]. In the intestine, no obvious morphological or cellular alterations accompany the delay in the functional enzymatic activity of epithelial cells in *Hoxa5^−^*^/*−*^ mutants [28]. In contrast, cell specification is perturbed in the gastric epithelium [27]. As for the lung and colon, goblet cell hyperplasia occurs in the stomach, strengthening the importance of *Hoxa5* for the correct specification of this secretory cell lineage. Concomitantly, less zymogenic cells, which release pepsinogen, and fewer enteroendocrine cells, a source of secretagogues stimulating pepsinogen production, are observed in the glandular epithelium, explaining the reduced pepsin enzymatic activity detected in *Hoxa5^−^*^/*−*^ mice. These changes in cell fate are compatible with a homeotic transformation of the hindstomach toward an intestine identity. They also concur with shifts in *Shh* and *Ihh* complementary expression domains along the gastric mucosa as well as with the altered expression of *Fgf10* and *Tgfßs*, leading to the model that *Hoxa5* provides regional cues that ensure the establishment of signaling networks warranting proper gut patterning [27].

### 2.5. Thyroid Gland

Surviving *Hoxa5* mutants present hypothyroidism symptoms, including growth retardation and delays in eye opening and ear elevation [29]. However, growth retardation cannot be attributed exclusively to hypothyroidism as the delayed acquisition of an adult mode of digestion that accompanies the impaired maturation of the digestive tract in *Hoxa5* mutants can also contribute to the growth deficit [28]. The lack of *Hoxa5* function perturbs the development and the structural organization of the thyroid gland at late gestation, as shown by the smaller and disorganized follicles and the large proportion of thyroglobulin-depleted follicles. *Hoxa5* expression is restricted to the mesenchyme adjoining the thyroid gland, the latter originating in part from the foregut as the lung and the stomach [29]. In *Hoxa5* mutants, the expression of *Nkx2-1*, *Pax8* and *Titf2* genes, key regulators of thyroid ontogeny and function, is altered suggesting that *Hoxa5* acts on thyroid development in a non-cell autonomous manner that requires mesenchyme-epithelium signaling.

### 2.6. Mammary Gland

Despite their transient hypothyroidism phenotype, surviving *Hoxa5* homozygous mice are fertile. However, *Hoxa5^−^*^/*−*^ females cannot feed properly their pups, due to abnormal post-natal mammary gland development [30]. Mammary glands develop under the control of a complex interplay involving systemic hormones, local growth factors and mesenchyme-epithelium communications [61]. The *Hoxa5* mutation causes inappropriate precocious mammary epithelium development with no major change in hormonal levels. Proliferation is augmented and accelerated differentiation occurs in nulliparous and pregnant females preceding the abnormal secretory activity at parturition that underlies the incapacity of *Hoxa5^−^*^/*−*^ dams to correctly nourish their progeny [30]. The accelerated lobuloalveolar epithelium development can be rescued upon grafting of mutant mammary epithelium into wild-type fat pads. Conversely, reciprocal grafting experiments demonstrate that *Hoxa5^−^*^/*−*^ stroma cannot support normal proliferation of wild-type epithelium, a result that is in accordance with the restricted expression of *Hoxa5* transcripts to the mammary stroma in mice. This unveils the importance of *Hoxa5* in the delicate balance between cell growth and differentiation and establishes the essential contribution of *Hoxa5* to mammary epithelium instruction via a mesenchyme-epithelium crosstalk.

### 2.7. Ovary

*Hoxa5^−^*^/*−*^ female mice also exhibit a precocious puberty and an early onset of estrous acyclicity that worsens with age, a phenotype coherent with an implication of *Hoxa5* in ovarian biology [31]. *Hoxa5* is mainly expressed in the stroma of the ovary and oviduct throughout the estrous cycle and expression levels increase with age. *Hoxa5* transcripts are also detected during gestation with a progressive raise due to the increasing expression in the corpus lutea. This dynamic expression profile of *Hoxa5* suggests that it may be subjected to regulation by sexual hormones. It also proposes specific roles for *Hoxa5* according to the cell type and the hormonal status. 

The loss of *Hoxa5* function leads to ovarian epithelial cyst formation in older females, a phenotype reminiscent of human endosalpingiosis, a pelvic condition characterized by the presence of epithelium-lined cystic structures [62]. The ovarian epithelial cysts detected in *Hoxa5^−^*^/*−*^ females likely derive from the ovarian surface epithelium (OSE). OSE is a simple mesothelium surrounding the outer surface of the ovary that has a very plastic phenotype [63]. Following release of the oocyte during ovulation, OSE cells proliferate and cover the wound [64]. Invaginations of the epithelium result in crypts that can be pinched off to form inclusion cysts within the stroma [65]. In normal conditions, OSE cells trapped in the stroma undergo an epithelial-mesenchymal transition to become integrated into the ovarian stroma. Failure of OSE cells to switch to a fibroblast-like identity favors cyst formation [66]. The presence of ovarian epithelial cysts in *Hoxa5^−^*^/*−*^ females indicates that the loss of *Hoxa5* function in ovarian stroma may affect OSE cell behavior. This is supported by the decreased expression of proteins involved in EGFR signaling, a key pathway for ovarian homeostasis [67]. Thus, *Hoxa5* is necessary to warrant proper EGFR signaling that is essential for the harmonious postovulatory epithelium repair process and ovarian physiology.

### 2.8. Hematopoiesis

Finally, *Hoxa5* is associated with hematopoiesis [68,69]. *HOXA5* is expressed in human bone marrow progenitor cells. Removal of *HOXA5* expression inhibits the differentiation towards a granulocytic/monocytic cell fate and favors the proliferation of a population of multipotential cells of an erythroid-committed subtype. Conversely, sustained *HOXA5* expression promotes myelopoiesis and prevents erythropoiesis.

In summary, perturbed epithelium represents a common theme in the different organs affected by the *Hoxa5* mutation in mice. As *Hoxa5* expression is mainly restricted to the mesenchyme in lung, gut, thyroid, mammary gland and ovary, the control of mesenchymal-epithelial interactions appears as the *modus operandi* of *Hoxa5* action. These observations support the concept that *Hoxa5* may govern similar pathways in organs undergoing related developmental processes. They also establish that *Hoxa5* is a key developmental regulator in organogenesis.

## 3. *Hoxa5*: Deregulated Expression and Tumorigenesis

The ability of *Hox* genes to control morphogenesis implies their role in multiple cellular processes. Since aberrant proliferation, survival, differentiation, adhesion, and migration are hallmarks of cancer cells, it is not surprising that deregulated *Hox* expression is associated with oncogenesis. Several studies have revealed the potential role of *Hox* genes in tumor development, invasion and metastasis. In numerous types of cancer, expression of specific *Hox* genes is either increased or decreased, suggesting that they may be involved in tumor promotion or suppression [70,71]. Changes in *Hox* gene expression in various cancers have been associated with altered proliferation, angiogenesis, apoptosis, DNA repair and metastatic behavior [72,73,74,75]. *Hox* misregulation can perturb the expression of downstream effectors, causing improper activation of embryonic developmental cascade(s), thereby disrupting normal programs of growth and differentiation and leading to neoplasia. Increased incidence of malignancies also correlates with ectopic cervical ribs in humans, a frequent skeletal transformation found in *Hox* mutant mice. Thus, *Hox* genes may be the molecular link between congenital anomalies and cancer [76].

*Hoxa5^−^*^/*−*^ mice are not prone to spontaneous tumorigenesis, indicating that the loss of *Hoxa5* function is not a genetic lesion sufficient to initiate oncogenesis. In normal human breast tissue, the HOXA5 protein is detected in ductal epithelial, myoepithelial and stromal cells at the parous, nulliparous and post-menopausal stages. Expression levels are low during lactation [77]. Nearly 70% of human breast carcinomas have decreased HOXA5 protein levels compared to normal tissue. Moreover, the loss of *HOXA5* gene expression in human breast cancer correlates with progression to higher-grade lesions, suggesting that it may act as a tumor suppressor gene [77,78]. In breast cancer cell lines and patient tumors, *HOXA5* silencing was proposed to result from the methylation of CpG islands in the *HOXA5* promoter region [78]. Reduced *HOXA5* expression is also associated with decreased *p53* expression. Transactivation and electrophoretic mobility shift assays showing a direct binding of the HOXA5 protein to a putative HOX-binding motif in the *p53* promoter support the idea that HOXA5 may possess growth-suppressive properties through activation of *p53* expression and apoptosis [78]. HOXA5 was also shown to interact with the transcription factor TWIST in breast cancer cell lines [79]. When overexpressed, TWIST demonstrates oncogenic potential by compromising the p53 response and cell cycle progression. HOXA5 can reverse the p53-suppressive effects of TWIST, acting as a safeguard of the p53 response via its ability to augment *p53* expression and by its capacity to bind TWIST thus limiting its negative action on p53. Alternatively, HOXA5 can induce cell death through a p53-independent program involving caspases 2 and 8 [80]. HOXA5 may also have an indirect effect on the integrity of the genome by regulating the expression of the mismatch repair gene *hMLH1* [81].

Although *Hoxa5^−^*^/*−*^ mice do not develop mammary tumors, the epithelial mispecification and the hyperplasia seen in the mammary glands of *Hoxa5^−^*^/*−*^ females suggest a protective role for *Hoxa5* toward cancer predisposition and reinforce the notion that *Hoxa5* may possess tumor-suppressive properties [30]. In *Hoxa5*;*p53* compound mutant mice, the presence of *Hoxa5* null alleles increases the susceptibility of *p53*^−/−^ mice to develop tumors with a higher prevalence for thymic lymphomas [82]. Grafting experiments of whole mammary glands from *Hoxa5*;*p53* compound mutants into wild-type recipients reveal that the loss of both *Hoxa5* functional alleles in *p53*^+/−^ mammary grafts triggers mammary tumor development, establishing the cooperative action of *Hoxa5* and *p53* in mammary tumorigenesis. However, this collaboration does not imply *p53* misregulation, which may reflect differences between species [82].

As mentioned, the ovarian epithelial inclusion cysts detected in *Hoxa5^−^*^/*−*^ females likely originate from the OSE. Although usually benign, OSE-derived inclusion cysts are thought to be a potential source of ovarian cancer and their more frequent occurrence in women with hereditary risk of ovarian cancer strengthens this hypothesis [83]. Human epithelial inclusion cysts express proteins detected in ovarian cancers, such as E-cadherin, p53, c-KIT, HER-2/neu, PAX8 and WT1, consistent with the hypothesis that they are preneoplastic lesions [84,85,86,87,88,89]. The ovarian epithelial inclusion cysts in *Hoxa5^−^*^/*−*^ mice also express the ovarian cancer markers PAX8 and WT1 suggesting that they constitute preneoplastic lesions and that the loss of *Hoxa5* function confers ovarian cancer predisposition [31]. 

Reduced *HOXA5* expression is a biomarker for poor prognostic in human non-small cell lung cancer (NSCLC). Indeed, HOXA5 controls NSCLC cell proliferation by positively regulating the expression of *Cdkn1a*, encoding the cyclin-dependent kinase inhibitor p21 [90]. Downregulation of *HOXA5* gene in NSCLC can occur due to aberrant promoter methylation or following *HOXA5* suppression by the microRNA-196a, which directly binds the 3′ untranslated region (UTR) of the *HOXA5* transcript [55,91].

A progressive downregulation of HOXA5 expression is observed from normal colon tissue to adenoma to carcinoma [92]. The decreased HOXA5 expression in colorectal tumors correlates with high levels of nuclear β-catenin, a hallmark of Wnt signaling activity. Moreover, *HOXA5* overexpression in colorectal tumors following a retinoid treatment suppresses the self-renewal capacity of cancer stem cells by inhibiting Wnt signaling. It also induces epithelial differentiation and reduces tumor size and metastatic incidence [92]. Interestingly, the negative action of *Hoxa5* on the Wnt canonical pathway was also reported in the developing lungs, reinforcing the notion that *Hoxa5* is a critical regulator of the Wnt signaling cascade [24].

*HOXA5* is expressed in quiescent endothelial cells, but absent in angiogenic endothelial cells. Forced expression of HOXA5 in activated angiogenic endothelial cells was reported to prevent angiogenesis by inhibiting the expression of pro-angiogenic molecules, like the VEGF receptor 2, while promoting the expression of the anti-angiogenic factor Thrombospondin-2 [93]. *HOXA5* downregulation in endothelial cells is mediated by the microRNA-130a that specifically targets a consensus sequence in the 3′-UTR of the *HOXA5* transcript [94]. Altogether, these data raise the possibility that HOXA5 may become a potential novel therapeutic agent limiting tumor progression.

In all previous cases, decreased *HOXA5* expression is associated with tumorigenesis. Involvement of *Hoxa5* in cancer was also revealed in acute leukemias induced by the translocation t(10;11)(p13;q14) that produces the fusion of the *AF10* gene, encoding a transcription factor, with the *CALM* (Clathrin Assembly Lymphoid Myeloid) leukemia gene [95]. The leukemic transformation was shown to require *Hoxa5* upregulation through the methylation of lysine 79 on histone H3 (H3K79) at the *Hoxa5* locus by hDOT1L, a methyltransferase that interacts with the CALM-AF10 fusion protein via its AF10 moiety [96]. In bone marrow cells from *Hoxa5^−^*^/*−*^ mice, the CALM-AF10 fusion protein cannot cause transformation. Thus, *Hoxa5* overexpression is critical for CALM-AF10-mediated leukemic transformation. Moreover, this does not appear to be limited to leukemia involving CALM-AF10 as *Hoxa5* upregulation was observed in other leukemic cell lines [97].

In summary, in all organs affected in *Hoxa5* mutant mice, perturbed cell proliferation and impaired differentiation are common denominators, indicating that *Hoxa5* resides at a key position in the cell growth and differentiation hierarchies. The same situation prevails in tumors. Thus, either a gain or a loss of *Hoxa5* gene expression may disrupt normal growth and differentiation programs causing neoplasia. It now remains to be determined how *Hoxa5* action is mediated by identifying its direct and indirect downstream effectors. In addition to the HOXA5 transcriptional targets mentioned above (the tumor suppressor gene *p53*, the mismatch repair gene *MutL homolog 1* and the follistatin-like 1 gene *Fstl1*), reported HOXA5 targets include the genes encoding the progesterone receptor and the cytokine pleiotrophin [98,99]. All these data also raise the possibility that acting on *Hoxa5* expression levels could provide a potent therapeutic strategy.

## 4. *Hoxa5*: Transcriptional Complexity and Mechanistic Integration

*Hox* function is intimately linked to the correct developmental expression of *Hox* genes as illustrated by the homeotic transformations observed when *Hox* *cis*-acting regulatory elements are mutated [100,101,102]. The critical role of *Hox* genes in development is clearly recognized but many gaps remain regarding the mechanisms that tightly control the establishment and maintenance of *Hox* gene expression in a precise spatio-temporal fashion in the embryo. A complex array of modes of regulation governs *Hox* gene expression [103,104]. Regulation primarily occurs at the transcriptional level via the combinatorial interplay of several signaling pathways and transcriptional factors that interact with positive and negative *cis*-acting sequences to differentially control *Hox* expression in a spatio-temporal and tissue-specific manner. The proximity of *Hox* genes in the clusters implies the integrated regulation of adjacent *Hox* promoters through the sharing, the competition and/or the selective use of defined enhancers [105]. Global regulatory elements located outside the *Hox* clusters are able of long-distance action to coordinate the expression of several genes along the *Hox* complexes [106]. Large-scale chromatin remodeling events also participate to the regulation of the collinear expression of *Hox* genes [107]. Finally, the discovery of non-coding RNAs throughout *Hox* clusters has unveiled additional levels of regulation of *Hox* expression implicating epigenetic control [108,109,110].

**Figure 1:**
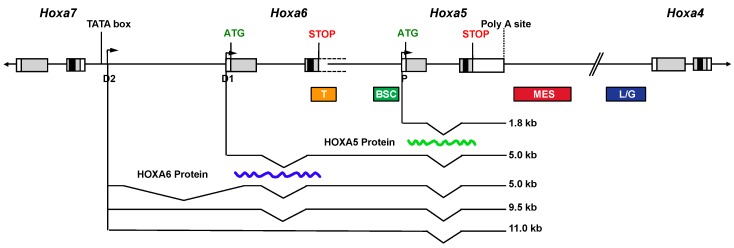
Schematic representation of the transcripts encompassing *Hoxa5* sequences and of the regulatory regions of the *Hoxa5* proximal promoter in the mouse embryo. Genomic organization of the *Hoxa4*, *Hoxa5*, *Hoxa6* and *Hoxa7* genes along the *HoxA* cluster. Black, grey and open boxes indicate homeobox, translated and transcribed sequences, respectively. The two known exons of *Hoxa5* and the start codon ATG of the HOXA5 protein are represented. The 3′ non-coding sequences of *Hoxa6* exon 2 extend further downstream into the *Hoxa6*-*Hoxa5* intergenic region and they are indicated by dotted lines. The start codon ATG of the putative HOXA6 protein is indicated. The promoters driving expression of the different transcripts are shown: proximal promoter, P; distal promoters D1 and D2. The transcripts are represented underneath. The waved lines correspond to the translated HOXA5 and HOXA6 proteins. Colored boxes define the DNA control sequences regulating *Hoxa5* expression driven by the P promoter. T, temporal; BSC, brachial spinal cord; MES, mesodermal enhancer sequence; L/G, Lung and gut. (Adapted from [19]).

A complex organization of overlapping transcriptional units encompassing the *Hoxa5* locus exists in the mouse embryo, which results from alternative splicing and the use of three promoters: one proximal producing the 1.8-kb transcript and two distal ones (D1 and D2) giving rise to four long RNAs (5.0- (2×), 9.5- and 11.0-kb transcripts; Figure 1). The distal promoter D1 corresponds to the putative *Hoxa6* promoter, while the most distal one (D2) is located in the *Hoxa6*–*Hoxa7* intergenic region downstream the *Hoxa7* gene [19]. The D1 and D2 putative promoters possess *Hox*-like transcriptional activity as shown by transgenesis. Sequence comparison of the D2 promoter region reveals a highly conserved DNA sequence of 160-bp, including a putative TATA box and a transcription start site (TSS), thus arguing for important evolutionary conserved regulatory DNA elements involved in the production of the larger transcripts. The 5.0-, 9.5- and 11.0-kb transcripts are expressed later and in more posterior embryonic structures than the 1.8-kb transcript, which recapitulates the *Hoxa7* expression domain [19,111]. All the *Hoxa5*-associated transcripts include the ORF encoding the HOXA5 protein but only the 1.8-kb form produces the protein, which is restricted to the cervico-thoracic region, where solely the 1.8-kb transcript is expressed. However, the 5-kb *Hoxa6/a5* transcript can produce the HOXA6 protein as shown by transfection assays in HEK293 cells. Thus, the 5-kb *Hoxa5*, the 9.5-kb and the 11.0-kb transcripts, all transcribed from the D2 promoter, can be considered as long non-coding (lnc) RNAs. The characterization of *Hoxa6*^−/−^ mutant embryos shows that the *Hoxa6* null mutation does not preclude the transcription of the 5.0-, 9.5- and 11.0-kb *Hoxa5* lncRNAs but it causes the production of 1-kb larger mutant transcripts due to the presence of the *neo* cassette [18]. This modification of the lncRNAs does not result in phenotypic consequences in the posterior domain of the embryo where they are expressed indicating that an alteration of the *Hoxa5* lncRNA sequences does not trigger major physiological effects [19]. The functional significance of the *Hoxa5*-associated lncRNAs remains to be determined. LncRNAs are present along the *Hox* clusters, some participating to the epigenetic regulation of *Hox* expression. LncRNAs can control negatively or positively gene expression acting either in *cis* or in *trans* [109,112,113]. All these data refute the initial view that lncRNAs correspond to transcriptional noise.

The presence of complex and overlapping transcriptional units at the *Hoxa5* locus implies dispersed and shared regulatory regions in the cluster. Even though the *Hoxa5* null mutation perturbs all *Hoxa5* transcripts [15], it only affects regions that express the 1.8-kb transcript, indicating that the 1.8-kb transcript is the functional form of the *Hoxa5* gene. Hence, studies combining transgenesis and molecular approaches were done to identify the regulatory elements of the *Hoxa5* proximal promoter (Figure 1). A 14.5-kb genomic fragment, starting within the neighboring *Hoxa6* gene and containing sequences up to the *Hoxa4* gene, largely reproduces the *Hoxa5* spatio-temporal expression driven by the proximal promoter in mouse embryos [114]. It encompasses *cis*-acting DNA control elements, such as a 604-bp brachial spinal cord (BSC) enhancer and a 650-bp temporal control region, both contained in the *Hoxa5* 5′-flanking sequences [115,116,117,118]. A 2.1-kb mesodermal enhancer sequence (MES) situated downstream the *Hoxa5* coding sequences was shown to be essential for *Hoxa5* paraxial and lateral plate mesoderm expression in the cervical and upper thoracic region [118]. The MES includes DNA elements that limit the *Hoxa5* regional specific expression domain along the anterior-posterior axis, including a 164-bp DNA sequence that binds CDX proteins to correctly position the *Hoxa5* expression domain [119]. A 1.5-kb DNA region that targets *Hoxa5* lung and gut developmental expression was also identified in the *Hoxa4*-*Hoxa5* intergenic sequences [120]. The 1.5-kb sequence includes a retinoic acid response element (RARE). *Hox* genes, mainly from paralog groups 1 to 5, are directly responsive to retinoic acid (RA), which activates retinoic acid receptors that then interact with RARE identified near *Hox* genes [101,121]. The identified RARE sequence is important for the establishment of the correct *Hoxa5* expression domain in the neural tube. However, despite the fact that it was reported to be necessary to drive *Hoxa4* expression in embryonic lung and gut, this RARE site does not play a key role in *Hoxa5* lung and gut expression during development [114]. Thus, the action of the RARE in organogenesis appears to be *Hox* promoter-specific [122]. Another RARE located at the 3′ end of the human *HOXA5* gene and conserved in the mouse genome was shown to mediate RA responsiveness of *HOXA5* in breast cancer cells [123].

The 1.5-kb *Hoxa5* lung/gut-specific *cis*-acting enhancer also interacts with the ubiquitous zinc-finger-containing transcription factor YY1. YY1 contains diverse domains enabling a plethora of protein-protein interactions. It can recruit coactivators or corepressors, which determine whether YY1 will execute inhibitory or activating functions on targets [124]. Even though the role of YY1 in *Hox* regulation was mainly associated with gene repression, YY1 acts as a positive regulator of *Hoxa5* gene expression in the developing respiratory and digestive tracts [114]. Moreover, the conditional deletion of *Yy1* function in lung mesenchyme results in mutant mice presenting a *Hoxa5*-like lung phenotype with decreased *Hoxa5* and *Hoxa4* gene expression. Therefore, the regulation driven by the YY1 binding sites located in the lung/gut enhancer in the intergenic *Hoxa5*-*Hoxa4* region is shared between the *Hoxa5* and *Hoxa4* genes. Interestingly, the specific ablation of *Yy1* in the lung epithelium impairs lung branching and causes airway dilation similar to that seen in pleuropulmonary blastoma, a rare pediatric lung cancer [125]. This reveals how critical YY1 is for lung morphogenesis. YY1 binding sites are also found in *Hoxa5* upstream sequences. Upon binding to these sites, YY1 mediates Polycomb Group (PcG) repression of *Hoxa5* expression in the anterior domain of the trunk, participating in the establishment of the correct *Hoxa5* expression domain in the prevertebral column [126]. Thus, depending on the developmental context, YY1 can mediate repression or activation of *Hoxa5* gene expression.

DNA methylation contributes to *Hoxa5* gene expression. In the mouse, *Hoxa5* exhibits a tissue-specific pattern of methylation of CpG islands in the promoter region that is established postnatally. The extent of *Hoxa5* methylation negatively correlates with the expression levels in adult tissues, suggesting that DNA methylation may participate in the temporal control of *Hoxa5* activity after birth [127]. Several microRNAs have also been shown to mediate *Hoxa5* downregulation [55,94,128,129,130,131]. miR-130a is frequently cited to contribute to *Hoxa5* post-transcriptional regulation. For example, RA treatment decreases miR-130a levels, which derepress HOXA5 translation. The induction of HOXA5 upon RA addition is also mediated by the RNA binding protein Human antigen R (HuR), which binds the 3′-UTR of *HOXA5* transcript to increase its stability [129]. Thus, RA modulates *Hoxa5* expression at the transcriptional and post-transcriptional levels. Not surprisingly, changes in the epigenetic control of *Hoxa5* have profound effects on its expression that are often linked to cancer. In some instances, *HOXA5* hypermethylation correlates with *HOXA5* downregulation and tumor progression [91,132,133]. As well, overexpression of miRNAs that target *Hoxa5* inhibits its expression and favors tumorigenesis [55,94,130,131].

## 5. Conclusion

The data accumulated over the years on the *Hoxa5* gene function clearly demonstrate that *Hoxa5* occupies a critical position in the developmental hierarchy that governs embryo patterning and organogenesis. Despite that, several questions remain. We still have to elucidate which signals transduce the information produced during early embryogenesis to correctly trigger *Hoxa5* expression in time and space and the regulatory mechanisms that maintain it throughout development. It is also essential to decode how *Hoxa5* provides regional cues to the developing organs and tissues by identifying the effectors and gene regulatory networks that mediate *Hoxa5* functions. Deciphering *Hoxa5* modes of action during development will provide insights into the molecular etiology of developmental and adult pathologies that may uncover novel therapeutic approaches.

## Figures and Tables

**Table 1:** *Hoxa5* mutant phenotypes.

Organs/Structures		Mutant Phenotypes	References
Axial skeleton		*Homeotic transformations:* *C6* → *C5, loss of tuberculum anterior* *C7* → *T1, ectopic ribs* *T1* → *T2, gain of dorsal process on T1* *T2* → *T1, loss of dorsal process on T2* *Sternum malformations:* Fusion of cervical ribs to the manubrium sterni Malformed xiphoid process Disorganized sternebrae	[15,16]
Appendicular skeleton		*Reduced/interrupted/missing acromion*	[16,20]
Respiratory system	Tracheal mesenchyme	*Reduced luminal surface to complete occlusion* *Thickening of the lamina propria* *Abnormal patterning of cartilage rings*	[21,22]
	Tracheal epithelium	*Epithelium disorganization:* Pseudostratified → stratified epithelium	[21]
	Lung mesenchyme	*Lung hypoplasia:* Reduced cell proliferation Reduced branching morphogenesis *Disorganization of the lung mesenchyme:* Abnormal compact appearance/Thicker mesenchyme Narrower airspaces in embryo *Impaired motility of alveolar myofibroblast precursors:* Mispositioning of the alveolar myofibroblasts Elastic fiber disorganization and altered alveogenesis Lung airspace enlargement in adult	[21,22,23]
	Lung epithelium	*Decreased surfactant protein expression* *Reduced number of secretory club cells* *Reduced number of alveolar type I pneumocytes* *Goblet cell metaplasia and mucus hypersecretion*	[21,22,23,24]
	Lung endothelium	*Undeveloped lung microvasculature:* Less and trapped endothelial cells within the dense mesenchyme	[22]
	Phrenic motor column	*Impaired diaphragm innervation*	[22,25]
	Respiratory function	*Impaired breathing:* Increased upper airway resistance Increased breathing frequency and overall minute ventilation in resting conditions Adaptation of the tidal volume and breathing frequency to maintain a higher minute ventilation when facing hypoxia	[26]
Digestive system	Stomach	*Perturbed cell specification of the gastric epithelium* *Goblet cell hyperplasia* *Reduced number of zymogenic and enteroendocrine cells:* Reduced pepsin enzymatic activity	[27]
	Intestine	*No obvious morphological defects* *Delay in the functional enzymatic activity of intestinal epithelial cells*	[28]
	Colon	*Abnormal distribution of goblet cells*	[27]
Thyroid gland		*Embryo:* Smaller and disorganized follicles Increased proportion of thyroglobulin-depleted follicles *Adult:* Hypothyroidism symptoms: Delayed eye opening and ear elevation Growth retardation	[29]
Mammary gland		*Abnormal development:* Mutant females cannot feed properly their pups Increased proliferation and accelerated differentiation of mammary epithelium	[30]
Ovary		*Precocious puberty* *Early onset of estrous acyclicity that worsens with age* *Ovarian epithelial cyst formation in older females*	[31]

## References

[1] Akam M. (1987). The molecular basis for metameric pattern in the Drosophila embryo. Development.

[2] Lewis E.B. (1978). A gene complex controlling segmentation in Drosophila. Nature.

[3] Krumlauf R. (1994). Hox genes in vertebrate development. Cell.

[4] Duboule D. (2007). The rise and fall of Hox gene clusters. Development.

[5] Tschopp P., Tarchini B., Spitz F., Zakany J., Duboule D. (2009). Uncoupling time and space in the collinear regulation of Hox genes. PLoS Genet..

[6] Mallo M., Wellik D.M., Deschamps J. (2010). Hox genes and regional patterning of the vertebrate body plan. Dev. Biol..

[7] Greer J.M., Puetz J., Thomas K.R., Capecchi M.R. (2000). Maintenance of functional equivalence during paralogous Hox gene evolution. Nature.

[8] Tvrdik P., Capecchi M.R. (2006). Reversal of Hox1 gene subfunctionalization in the mouse. Dev. Cell.

[9] Mann R.S., Lelli K.M., Joshi R. (2009). Hox specificity: Unique roles for cofactors and collaborators. Curr. Top. Dev. Biol..

[10] Rezsohazy R., Saurin A.J., Maurel-Zaffran C., Graba Y. (2015). Cellular and molecular insights into Hox protein action. Development.

[11] Pearson J.C., Lemons D., McGinnis W. (2005). Modulating Hox gene functions during animal body patterning. Nat. Rev. Genet..

[12] Quinonez S.C., Innis J.W. (2014). Human HOX gene disorders. Mol. Genet. Metab..

[13] Bhatlekar S., Fields J.Z., Boman B.M. (2014). HOX genes and their role in the development of human cancers. J. Mol. Med..

[14] Odenwald W.F., Taylor C.F., Palmer-Hill F.J., Friedrich V., Tani M., Lazzarini R.A. (1987). Expression of a homeo domain protein in noncontact-inhibited cultured cells and postmitotic neurons. Genes Dev..

[15] Jeannotte L., Lemieux M., Charron J., Poirier F., Robertson E.J. (1993). Specification of axial identity in the mouse: Role of the Hoxa-5 (Hox1. 3) gene. Genes Dev..

[16] Aubin J., Lemieux M., Tremblay M., Behringer R.R., Jeannotte L. (1998). Transcriptional interferences at the Hoxa4/Hoxa5 locus: Importance of correct Hoxa5 expression for the proper specification of the axial skeleton. Dev. Dyn..

[17] Horan G.S., Wu K., Wolgemuth D.J., Behringer R.R. (1994). Homeotic transformation of cervical vertebrae in Hoxa-4 mutant mice. Proc. Natl. Acad. Sci. USA.

[18] Kostic D., Capecchi M.R. (1994). Targeted disruptions of the murine Hoxa-4 and Hoxa-6 genes result in homeotic transformations of components of the vertebral column. Mech. Dev..

[19] Coulombe Y., Lemieux M., Moreau J., Aubin J., Joksimovic M., Bérubé-Simard F.A., Tabariès S., Boucherat O., Guillou F., Larochelle C. (2010). Multiple promoters and alternative splicing: Hoxa5 transcriptional complexity in the mouse embryo. PLoS ONE.

[20] Aubin J., Lemieux M., Moreau J., Lapointe J., Jeannotte L. (2002). Cooperation of Hoxa5 and Pax1 genes during formation of the pectoral girdle. Dev. Biol..

[21] Aubin J., Lemieux M., Tremblay M., Bérard J., Jeannotte L. (1997). Early postnatal lethality in Hoxa-5 mutant mice is attributable to respiratory tract defects. Dev. Biol..

[22] Boucherat O., Montaron S., Bérubé-Simard F.A., Aubin J., Philippidou P., Wellik D.M., Dasen J.S., Jeannotte L. (2013). Partial functional redundancy between Hoxa5 and Hoxb5 paralog genes during lung morphogenesis. Am. J. Physiol. Lung Cell Mol. Physiol..

[23] Mandeville I., Aubin J., LeBlanc M., Lalancette-Hébert M., Janelle M.F., Tremblay G.M., Jeannotte L. (2006). Impact of the loss of Hoxa5 function on lung alveogenesis. Am. J. Pathol..

[24] Boucherat O., Chakir J., Jeannotte L. (2012). The loss of Hoxa5 function promotes Notch-dependent goblet cell metaplasia in lung airways. Biol. Open.

[25] Philippidou P., Walsh C.M., Aubin J., Jeannotte L., Dasen J.S. (2012). Sustained Hox5 gene activity is required for respiratory motor neuron development. Nat. Neurosci..

[26] Kinkead R., Leblanc M., Gulemetova R., Lalancette-Hébert M., Lemieux M., Mandeville I., Jeannotte L. (2004). Respiratory adaptations to lung morphological defects in adult mice lacking Hoxa5 gene function. Pediatr. Res..

[27] Aubin J., Déry U., Lemieux M., Chailler P., Jeannotte L. (2002). Stomach regional specification requires Hoxa5-driven mesenchymal-epithelial signaling. Development.

[28] Aubin J., Chailler P., Ménard D., Jeannotte L. (1999). Loss of Hoxa5 gene function in mice perturbs intestinal maturation. Am. J. Physiol. Cell Physiol..

[29] Meunier D., Aubin J., Jeannotte L. (2003). Perturbed thyroid morphology and transient hypothyroidism symptoms in Hoxa5 mutant mice. Dev. Dyn..

[30] Garin É., Lemieux M., Coulombe Y., Robinson G.W., Jeannotte L. (2006). Stromal Hoxa5 function controls the growth and differentiation of mammary alveolar epithelium. Dev. Dyn..

[31] Gendronneau G., Boucherat O., Aubin J., Lemieux M., Jeannotte L. (2012). The loss of Hoxa5 function causes estrous acyclicity and ovarian epithelial inclusion cysts. Endocrinology.

[32] Grüneberg H. (1954). Genetical studies on the skeleton of the mouse. J. Genet..

[33] Balling R., Deutsch U., Gruss P. (1988). Undulated, a mutation affecting the development of the mouse skeleton, has a point mutation in the paired box of Pax 1. Cell.

[34] Wallin J., Wilting J., Koseki H., Fritsch R., Christ B., Balling R. (1994). The role of Pax-1 in axial skeleton development. Development.

[35] Bi W., Deng J.M., Zhang Z., Behringer R.R., de Crombrugghe B. (1999). Sox9 is required for cartilage formation. Nat. Genet..

[36] Chen J.W., Zahid S., Shilts M.H., Weaver S.J., Leskowitz R.M., Habbsa S., Aronowitz K.P.R., Chang Y., Pinnella Z., Holloway L. (2013). Hoxa-5 acts in segmented somites to regulate cervical vertebral morphology. Mech. Dev..

[37] Rancourt D.E., Tsuzuki T., Capecchi M.R. (1995). Genetic interaction between hoxb-5 and hoxb-6 is revealed by nonallelic noncomplementation. Genes Dev..

[38] McIntyre D.C., Rakshit S., Yallowitz A.R., Loken L., Jeannotte L., Capecchi M.R., Wellik D.M. (2007). Hox patterning of the vertebrate rib cage. Development.

[39] Desir A., Ghaye B. (2009). Congenital abnormalities of intrathoracic airways. Radiol. Clin. North Am..

[40] Geng Y., Dong Y., Yu M., Zhang L., Yan X., Sun J., Qiao L., Geng H., Nakajima M., Furuichi T. (2011). Follistatin-like 1 (Fstl1) is a bone morphogenetic protein (BMP) 4 signaling antagonist in controlling mouse lung development. Proc. Natl. Acad. Sci. USA.

[41] Bohinski R.J., di Lauro R., Whitsett J.A. (1994). The lung-specific surfactant protein B gene promoter is a target for thyroid transcription factor 1 and hepatocyte nuclear factor 3, indicating common factors for organ-specific gene expression along the foregut axis. Mol. Cell. Biol..

[42] Cardoso W.V. (1995). Transcription factors and pattern formation in the developing lung. Am. J. Physiol. Lung Cell Mol. Physiol..

[43] Kim N., Vu T.H. (2006). Parabronchial smooth muscle cells and alveolar myofibroblasts in lung development. Birth Defects Res. C. Embryo Today.

[44] Park K.S., Korfhagen T.R., Bruno M.D., Kitzmiller J.A., Wan H., Wert S.E., Khurana Hershey G.K., Chen G., Whitsett J.A. (2007). SPDEF regulates goblet cell hyperplasia in the airway epithelium. J. Clin. Invest..

[45] Park S.W., Verhaeghe C., Nguyenvu L.T., Barbeau R., Eisley C.J., Nakagami Y., Huang X., Woodruff P.G., Fahy J.V., Erle D.J. (2009). Distinct roles of FOXA2 and FOXA3 in allergic airway disease and asthma. Am. J. Respir. Crit. Care Med..

[46] Wan H., Kaestner K.H., Ang S.L., Ikegami M., Finkelman F.D., Stahlman M.T., Fulkerson P.C., Rothenberg M.E., Whitsett J.A. (2004). Foxa2 regulates alveolarization and goblet cell hyperplasia. Development.

[47] Chen G., Korfhagen T.R., Xu Y., Kitzmiller J., Wert S.E., Maeda Y., Gregorieff A., Clevers H., Whitsett J.A. (2009). SPDEF is required for mouse pulmonary goblet cell differentiation and regulates a network of genes associated with mucus production. J. Clin. Invest..

[48] Guseh J.S., Bores S.A., Stanger B.Z., Zhou Q., Anderson W.J., Melton D.A., Rajagopal J. (2009). Notch signaling promotes airway mucous metaplasia and inhibits alveolar development. Development.

[49] Hrycaj S.M., Dye B.R., Baker N.C., Larsen B.M., Burke A.C., Spence J.R., Wellik D.M. (2015). Hox5 genes regulate the Wnt2/2b-Bmp4-signaling axis during lung development. Cell Rep..

[50] Gaunt S.J., Coletta D., Pravtcheva D., Sharpe P.T. (1990). Mouse Hox-3.4: Homeobox sequence and embryonic expression patterns with other members of the Hox gene gen network. Development.

[51] Liggins G.C., Vilos G.A., Campos G.A., Kitterman J.A., Lee C.H. (1981). The effect of spinal cord transection on lung development in fetal sheep. J. Dev. Physiol..

[52] Inanlou M.R., Baguma-Nibasheka M., Kablar B. (2005). The role of fetal breathing-like movements in lung organogenesis. Histol. Histopathol..

[53] Golpon H.A., Geraci M.W., Moore M.D., Miller H.L., Miller G.J., Tuder R.M., Voelkel N.F. (2001). HOX genes in human lung: Altered expression in primary pulmonary hypertension and emphysema. Am. J. Pathol..

[54] Boucherat O., Franco-Montoya M.L., Thibault C., Incitti R., Chailley-Heu B., Delacourt C., Bourbon J.R. (2007). Gene expression profiling in lung fibroblasts reveals new players in alveolarization. Physiol. Genomics.

[55] Liu X.H., Lu K.H., Wang K.M., Sun M., Zhang E.B., Yang J.S., Yin D.D., Liu X.L., Zhou J., Liu Z.J. (2012). MicroRNA-196a promotes non-small cell lung cancer cell proliferation and invasion through targeting HOXA5. BMC Cancer.

[56] Shi W., Warburton D. (2010). Is COPD in adulthood really so far removed from early development?. Eur. Respir. J..

[57] Grapin-Botton A., Melton D.A. (2000). Endoderm development: From patterning to organogenesis. Trends Genet..

[58] Roberts D.J. (2000). Molecular mechanisms of development of the gastrointestinal tract. Dev. Dynam..

[59] Dony C., Gruss P. (1987). Specific expression of the Hox 1.3 homeo box gene in murine embryonic structures originating from or induced by the mesoderm. EMBO J..

[60] James R., Kazenwadel J. (1991). Homeobox gene expression in the intestinal epithelium of adult mice. J. Biol. Chem..

[61] Hennighausen L., Robinson G.W. (2001). Signaling pathways in mammary gland development. Dev. Cell.

[62] Ryuko K., Miura H., Abu-Musa A., Iwanari O., Kitao M. (1992). Endosalpingiosis in association with ovarian surface papillary tumor of borderline malignancy. Gynecol. Oncol..

[63] Auersperg N., Wong A.S., Choi K.C., Kang S.K., Leung P.C. (2001). Ovarian surface epithelium: Biology, endocrinology, and pathology. Endocr. Rev..

[64] Murdoch W.J., McDonnel A.C. (2002). Roles of the ovarian surface epithelium in ovulation and carcinogenesis. Reproduction.

[65] Nicosia S.V. (1987). The aging ovary. Med. Clin. North Am..

[66] Auersperg N. (2011). The origin of ovarian carcinomas: A unifying hypothesis. Int. J. Gynecol. Pathol..

[67] Ahmed N., Maines-Bandiera S., Quinn M.A., Unger W.G., Dedhar S., Auersperg N. (2006). Molecular pathways regulating EGF-induced epithelio-mesenchymal transition in human ovarian surface epithelium. Am. J. Physiol. Cell Physiol..

[68] Fuller J.F., McAdara J., Yaron Y., Sakaguchi M., Fraser J.K., Gasson J.C. (1999). Characterization of HOX gene expression during myelopoiesis: Role of HOXA5 in lineage commitment and maturation. Blood.

[69] Crooks G.M., Fuller J., Petersen D., Izadi P., Malik P., Pattengale P.K., Kohn D.B., Gasson J.C. (1999). Constitutive HOXA5 expression inhibits erythropoiesis and increases myelopoiesis from human hematopoietic progenitors. Blood.

[70] Abate-Shen C. (2002). Deregulated homeobox gene expression in cancer: cause or consequence?. Nat. Rev. Cancer.

[71] Shah N., Sukumar S. (2010). The Hox genes and their roles in oncogenesis. Nat. Rev. Cancer.

[72] Carrio M., Arderiu G., Myers C., Boudreau N.J. (2005). Homeobox D10 induces phenotypic reversion of breast tumor cells in a three-dimensional culture model. Cancer Res..

[73] Rhoads K., Arderiu G., Charboneau A., Hansen S.L., Hoffman W., Boudreau N. (2005). A role for HoxA5 in regulating angiogenesis and vascular patterning. Lymphat. Res. Biol..

[74] Rubin E., Wu X., Zhu T., Cheung J.C., Chen H., Lorincz A., Pandita R.K., Sharma G.G., Ha H.C., Gasson J. (2007). A role for the HOXB7 homeodomain protein in DNA repair. Cancer Res..

[75] Chen H., Lee J.S., Liang X., Zhang H., Zhu T., Zhang Z., Taylor M.E., Zahnow C., Feigenbaum L., Rein A. (2008). Hoxb7 inhibits transgenic HER-2/neu–induced mouse mammary tumor onset but promotes progression and lung metastasis. Cancer Res..

[76] Anbazhagan R., Raman V. (1997). Homeobox genes: Molecular link between congenital anomalies and cancer. Eur. J. Cancer.

[77] Henderson G.S., van Diest P.J., Burger H., Russo J., Raman V. (2006). Expression pattern of a homeotic gene, HOXA5, in normal breast and in breast tumors. Cell. Oncol..

[78] Raman V., Martensen S.A., Reisman D., Evron E., Odenwald W.F., Jaffee E., Marks J., Sukumar S. (2000). Compromised HOXA5 function can limit p53 expression in human breast tumours. Nature.

[79] Stasinopoulos I.A., Mironchik Y., Raman A., Wildes F., Winnard P., Raman V. (2005). HOXA5-twist interaction alters p53 homeostasis in breast cancer cells. J. Biol. Chem..

[80] Chen H., Chung S., Sukumar S. (2004). HOXA5-induced apoptosis in breast cancer cells is mediated by caspases 2 and 8. Mol. Cell. Biol..

[81] Duriseti S., Winnard P.T., Mironchik Y., Vesuna F., Raman A., Raman V. (2006). HOXA5 regulates hMLH1 expression in breast cancer cells. Neoplasia.

[82] Gendronneau G., Lemieux M., Morneau M., Paradis J., Têtu B., Frenette N., Aubin J., Jeannotte L. (2010). Influence of Hoxa5 on p53 tumorigenic outcome in mice. Am. J. Pathol..

[83] Salazar H., Godwin A.K., Daly M.B., Laub P.B., Hogan W.M., Rosenblum N., Boente M.P., Lynch H.T., Hamilton T.C. (1996). Microscopic benign and invasive malignant neoplasms and a cancer-prone phenotype in prophylactic oophorectomies. J. Natl. Cancer Inst..

[84] Wang D.P., Konishi I., Koshiyama M., Nanbu Y., Iwai T., Nonogaki H., Mori T., Fujii S. (1992). Immunohistochemical localization of c-erbB-2 protein and epidermal growth factor receptor in normal surface epithelium, surface inclusion cysts, and common epithelial tumours of the ovary. Virchows Arch. A Pathol. Anat. Histopathol..

[85] Hutson R., Ramsdale J., Wells M. (1995). p53 protein expression in putative precursor lesions of epithelial ovarian cancer. Histopathology.

[86] Sundfeldt K., Piontkewitz Y., Ivarsson K., Nilsson O., Hellberg P., Brännström M., Janson P.O., Enerback S., Hedin L. (1997). E-cadherin expression in human epithelial ovarian cancer and normal ovary. Int. J. Cancer.

[87] Tonary A.M., Macdonald E.A., Faught W., Senterman M.K., Vanderhyden B.C. (2000). Lack of expression of c-KIT in ovarian cancers is associated with poor prognosis. Int. J. Cancer.

[88] Bowen N.J., Logani S., Dickerson E.B., Kapa L.B., Akhtar M., Benigno B.B., McDonald J.F. (2007). Emerging roles for PAX8 in ovarian cancer and endosalpingeal development. Gynecol. Oncol..

[89] Madore J., Ren F., Filali-Mouhim A., Sanchez L., Köbel M., Tonin P.N., Huntsman D., Provencher D.M., Mes-Masson A.M. (2010). Characterization of the molecular differences between ovarian endometrioid carcinoma and ovarian serous carcinoma. J. Pathol..

[90] Zhang M.L., Nie F.Q., Sun M., Xia R., Xie M., Lu K.H., Li W. (2015). HOXA5 indicates poor prognosis and suppresses cell proliferation by regulating p21 expression in non-small cell lung cancer. Tumor Biol..

[91] Kim D.S., Kim M.J., Lee J.Y., Lee S.M., Choi J.Y., Yoon G.S., Na Y.K., Hong H.S., Kim S.G., Choi J.E. (2009). Epigenetic inactivation of Homeobox A5 gene in non-small cell lung cancer and its relationship with clinicopathological features. Mol. Carcinog..

[92] Ordóñez-Morán P., Dafflon C., Imajo M., Nishida E., Huelsken J. (2015). HOXA5 counteracts stem cell traits by inhibiting Wnt signaling in colorectal cancer. Cancer Cell.

[93] Cuevas I., Layman H., Coussens L., Boudreau N. (2015). Sustained endothelial expression of HoxA5 *in vivo* impairs pathological angiogenesis and tumor progression. PloS ONE.

[94] Chen Y., Gorski D.H. (2008). Regulation of angiogenesis through a microRNA (miR-130a) that down-regulates antiangiogenic homeobox genes GAX and HOXA5. Blood.

[95] Dreyling M.H., Martinez-Climent J.A., Zheng M., Mao J., Rowley J.D., Bohlander S.K. (1996). The t (10; 11)(p13; q14) in the U937 cell line results in the fusion of the AF10 gene and CALM, encoding a new member of the AP-3 clathrin assembly protein family. Proc. Natl. Acad. Sci. USA.

[96] Okada Y., Jiang Q., Lemieux M., Jeannotte L., Su L., Zhang Y. (2006). Leukaemic transformation by CALM–AF10 involves upregulation of Hoxa5 by hDOT1L. Nat. Cell. Biol..

[97] Quentmeier H., Dirks W.G., Macleod R.A., Reinhardt J., Zaborski M., Drexler H.G. (2004). Expression of HOX genes in acute leukemia cell lines with and without MLL translocations. Leuk. Lymphoma.

[98] Raman V., Tamori A., Vali M., Zeller K., Korz D., Sukumar S. (2000). HOXA5 regulates expression of the progesterone receptor. J. Biol. Chem..

[99] Chen H., Rubin E., Zhang H., Chung S., Jie C.C., Garrett E., Biswal S., Sukumar S. (2005). Identification of transcriptional targets of HOXA5. J. Biol. Chem..

[100] Gérard M., Chen J.Y., Gronemeyer H., Chambon P., Duboule D., Zakany J. (1996). *In vivo* targeted mutagenesis of a regulatory element required for positioning the Hoxd-11 and Hoxd-10 expression boundaries. Genes Dev..

[101] Dupé V., Davenne M., Brocard J., Dollé P., Mark M., Dierich A., Chambon P., Rijli F.M. (1997). *In vivo* functional analysis of the Hoxa-1 3′ retinoic acid response element (3′ RARE). Development.

[102] Zákány J., Gérard M., Favier B., Duboule D. (1997). Deletion of a HoxD enhancer induces transcriptional heterochrony leading to transposition of the sacrum. EMBO J..

[103] Mallo M., Alonso C.R. (2013). The regulation of Hox gene expression during animal development. Development.

[104] Montavon T., Soshnikova N. (2014). Hox gene regulation and timing in embryogenesis. Semin. Cell. Dev. Biol..

[105] Sharpe J., Nonchev S., Gould A., Whiting J., Krumlauf R. (1998). Selectivity, sharing and competitive interactions in the regulation of Hoxb genes. EMBO J..

[106] Spitz F., Duboule D. (2008). Global control regions and regulatory landscapes in vertebrate development and evolution. Adv. Genet..

[107] Chambeyron S., Bickmore W.A. (2004). Chromatin decondensation and nuclear reorganization of the HoxB locus upon induction of transcription. Genes Dev..

[108] Ponting C.P., Oliver P.L., Reik W. (2009). Evolution and functions of long noncoding RNAs. Cell.

[109] Rinn J.L., Kertesz M., Wang J.K., Squazzo S.L., Xu X., Brugmann S.A., Goodnough L.H., Helms J.A., Farnham P.J., Segal E. (2007). Functional demarcation of active and silent chromatin domains in human HOX loci by noncoding RNAs. Cell.

[110] Sessa L., Breiling A., Lavorgna G., Silvestri L., Casari G., Orlando V. (2007). Noncoding RNA synthesis and loss of Polycomb group repression accompanies the colinear activation of the human HOXA cluster. RNA.

[111] Püschel A.W., Balling R., Gruss P. (1990). Position-specific activity of the Hox1.1 promoter in transgenic mice. Development.

[112] Wang K.C., Yang Y.W., Liu B., Sanyal A., Corces-Zimmerman R., Chen Y., Lajoie B.R., Protacio A., Flynn R.A., Gupta R.A. (2011). A long noncoding RNA maintains active chromatin to coordinate homeotic gene expression. Nature.

[113] Li L., Liu B., Wapinski O.L., Tsai M.-C., Qu K., Zhang J., Carlson J.C., Lin M., fang F., Gupta R.A. (2013). Targeted disruption of Hotair leads to homeotic transformation and gene derepression. Cell Rep..

[114] Bérubé-Simard F.A., Prudhomme C., Jeannotte L. (2014). YY1 Acts as a Transcriptional Activator of Hoxa5. PLoS ONE.

[115] Zakany J., Tuggle C.K., Patel M.D., Nguyen-Huu M.C. (1988). Spatial regulation of homeobox gene fusions in the embryonic central nervous system of transgenic mice. Neuron.

[116] Tuggle C.K., Zakany J., Cianetti L., Peschle C., Nguyen-Huu M.C. (1990). Region-specific enhancers near two mammalian homeo box genes define adjacent rostrocaudal domains in the central nervous system. Genes Dev..

[117] Nowling T., Zhou W., Krieger K.E., Larochelle C., Nguyen-Huu M.C., Jeannotte L., Tuggle C.K. (1999). Hoxa5 gene regulation: A gradient of binding activity to a brachial spinal cord element. Dev. Biol..

[118] Larochelle C., Tremblay M., Bernier D., Aubin J., Jeannotte L. (1999). Multiple cis-acting regulatory regions are required for restricted spatio-temporal Hoxa5 gene expression. Dev. Dyn..

[119] Tabariès S., Lapointe J., Besch T., Carter M., Woollard J., Tuggle C.K., Jeannotte L. (2005). Cdx protein interaction with Hoxa5 regulatory sequences contributes to Hoxa5 regional expression along the axial skeleton. Mol. Cell. Biol..

[120] Moreau J., Jeannotte L. (2002). Sequence analysis of a Hoxa4-Hoxa5 intergenic region including shared regulatory elements. DNA Seq..

[121] Oosterveen T., van Vliet P., Deschamps J., Meijlink F. (2003). The direct context of a Hox retinoic acid response element is crucial for its activity. J. Biol. Chem..

[122] Packer A.I., Crotty D.A., Elwell V.A., Wolgemuth D.J. (1998). Expression of the murine Hoxa4 gene requires both autoregulation and a conserved retinoic acid response element. Development.

[123] Chen H., Zhang H., Lee J., Liang X., Wu X., Zhu T., Lo P.K., Zhang X., Sukumar S. (2007). HOXA5 acts directly downstream of retinoic acid receptor β and contributes to retinoic acid–induced apoptosis and growth inhibition. Cancer Res..

[124] Deng Z., Cao P., Wan M.M., Sui G. (2010). Yin Yang 1: A multifaceted protein beyond a transcription factor. Transcription.

[125] Boucherat O., Landry-Truchon K., Bérubé-Simard F.A., Houde N., Beuret L., Lezmi G., Foulkes W.D., Delacourt C., Charron J., Jeannotte L. (2015). Epithelial inactivation of Yy1 abrogates lung branching morphogenesis. Development.

[126] Kim S.Y., Paylor S.W., Magnuson T., Schumacher A. (2006). Juxtaposed Polycomb complexes co-regulate vertebral identity. Development.

[127] Hershko A.Y., Kafri T., Fainsod A., Razin A. (2003). Methylation of HoxA5 and HoxB5 and its relevance to expression during mouse development. Gene.

[128] Mujahid S., Nielsen H.C., Volpe M.V. (2013). MiR-221 and miR-130a regulate lung airway and vascular development. PLoS ONE.

[129] Yang F., Miao L., Mei Y., Wu M. (2013). Retinoic acid-induced HOXA5 expression is co-regulated by HuR and miR-130a. Cell. Signal..

[130] Lee D.H., Forscher C., di Vizio D., Koeffler H.P. (2015). Induction of p53-independent apoptosis by ectopic expression of HOXA5 in human liposarcomas. Sci. Rep..

[131] Wang Y., Xu L., Jiang L. (2015). miR-1271 promotes non-small-cell lung cancer cell proliferation and invasion via targeting HOXA5. Biochem. Biophys. Res. Commun..

[132] Watson R.E., Curtin G.M., Hellmann G.M., Doolittle D.J., Goodman J.I. (2004). Increased DNA methylation in the HoxA5 promoter region correlates with decreased expression of the gene during tumor promotion. Mol. Carcinog..

[133] Strathdee G., Sim A., Soutar R., Holyoake T.L., Brown R. (2006). HOXA5 is targeted by cell-type-specific CpG island methylation in normal cells and during the development of acute myeloid leukaemia. Carcinogenesis.

